# Hand in Hand: Public Endorsement of Climate Change Mitigation and Adaptation

**DOI:** 10.1371/journal.pone.0124843

**Published:** 2015-04-29

**Authors:** Adrian Brügger, Thomas A. Morton, Suraje Dessai

**Affiliations:** 1 Faculty of Business, Economics and Social Sciences, University of Bern, Bern, Switzerland; 2 College of Life and Environmental Sciences, University of Exeter, Exeter, United Kingdom; 3 Sustainability Research Institute and ESRC Centre for Climate Change Economics and Policy, School of Earth and Environment, University of Leeds, Leeds, United Kingdom; 4 Climate Change Impacts, Adaptation, and Mitigation Research Group, Faculty of Sciences, University of Lisbon, Lisbon, Portugal; US Army Engineer Research and Development Center, UNITED STATES

## Abstract

This research investigated how an individual’s endorsements of mitigation and adaptation relate to each other, and how well each of these can be accounted for by relevant social psychological factors. Based on survey data from two European convenience samples (*N* = 616 / 309) we found that public endorsements of mitigation and adaptation are strongly associated: Someone who is willing to reduce greenhouse gas emissions (mitigation) is also willing to prepare for climate change impacts (adaptation). Moreover, people endorsed the two response strategies for similar reasons: People who believe that climate change is real and dangerous, who have positive attitudes about protecting the environment and the climate, and who perceive climate change as a risk, are willing to respond to climate change. Furthermore, distinguishing between (spatially) proximal and distant risk perceptions suggested that the idea of portraying climate change as a proximal (i.e., local) threat might indeed be effective in promoting personal actions. However, to gain endorsement of broader societal initiatives such as policy support, it seems advisable to turn to the distant risks of climate change. The notion that “localising” climate change might not be the panacea for engaging people in this domain is discussed in regard to previous theory and research.

## Introduction

To respond to the challenges posed by climate change, societies around the world are faced with two related but separate strategies. The first strategy, *mitigation*, involves reducing the magnitude of future climate change by cutting greenhouse gas emissions (e.g., reduction of energy consumption) and enhancing greenhouse gas sinks (e.g., afforestation) [1]. However, because the planet is already committed to a certain level of climate change [2], it is also important to prepare for and deal with the negative consequences of climate change (e.g., protecting coastal zones from sea-level rise) as well as taking advantage of the positive consequences of climate change (e.g., growing wine in regions that were previously too cold for that purpose). This second response to climate change is referred to as *adaptation* [2].

Implementing these response strategies, however, is challenging not least because it requires endorsement from the general public. If public endorsement is weak and priorities lie elsewhere, then unpopular mitigation and adaptation measures will meet resistance. Understanding how members of the public view mitigation and adaptation is also important because the amount of individuals’ greenhouse gas emissions—and therefore the potential for emission reductions— is substantial [3]. Individual motivation is also paramount in terms of adaptation: the responsibility for many adaptive responses entirely lies with individuals. In short, if mitigation and adaptation are to be successful, both as individual and collective (i.e., policy) responses, it is important to understand what motivates people to endorse each of these strategies.

While climate change experts agree that both mitigation and adaptation are necessary, currently there is little knowledge about the relationship between people’s endorsement of mitigation and their endorsement of adaptation. Conceptually, three relationships between mitigation and adaptation can be postulated: (1) a negative relationship, where the public considers mitigation and adaptation as competing alternatives—that is, people favour either mitigation or adaptation and discard the other; (2) a positive relationship, where people similarly endorse (or oppose) mitigation and adaptation, or; (3) there is no systematic relationship between endorsements for mitigation and adaptation.

The first possibility, a *negative relationship* between the two response strategies, mirrors how interest in mitigation and adaptation developed over time. Until recently, researchers and policy-makers almost exclusively focused on mitigation [4,5]. One of the reasons for the prioritising of mitigation was that focusing on adaptation evoked negative associations such as being “defeatist” or not willing to act [5]. Members of the public might take a similar stance and favour mitigation over adaptation.

In addition to the historical development, there are important differences between mitigation and adaptation that, on their own, could create a divide between endorsements of the two response strategies. The most striking difference is probably the temporal and spatial scale at which the two strategies work [4,6]. Mitigation requires immediate action but—due to the inertia of the climate system—it will take decades before mitigation efforts will show their benefits. To illustrate, it would take several decades before emission reductions stabilised the climatic changes that are already under way. In contrast, adaptation measures typically focus on short or medium term problems and often yield immediate benefits. An example that illustrates this is the upgrade of flood defences: once they are in place, they protect people to a higher standard of flood risk. With regard to space, effective mitigation must be implemented globally and will also have global benefits while adaptation efforts and benefits are constrained to the regional or local level [4]. There are a number of individual differences that could lead people to more strongly support early versus late action or (spatially) proximal versus distant solutions (e.g., the extent to which people are attached to proximal versus distant places [7]).

Despite the historical development and incontestable differences between mitigation and adaptation, a positive relationship, that is, *joint endorsement of* (or opposition to) the two strategies, is not implausible. After all, their common goal is to avoid negative consequences for the human and natural environment. If people’s endorsements of mitigation and adaptation are guided by a broader perspective that integrates both strategies as a common response to threats associated with climate change, then people who endorse one strategy, for example because they value nature, should also endorse the second strategy.

Lastly, there are at least two reasons why the relationship between mitigation and adaptation may be *unsystematic*. First, the media typically frame climate change mitigation and adaptation independently of each other [8]. This makes it difficult for laypeople to relate the two strategies to each other, other than via their common association with the issue of climate change. Second, it is possible that the way different people relate mitigation to adaptation varies strongly (e.g., some people may endorse both strategies because of their common goal, others favouring one strategy because of its specific characteristics). In this case the pattern between the two strategies could look unsystematic (i.e., zero-relationship). In sum, good reasons can be advanced for a negative, positive, or unsystematic relationship between mitigation and adaptation.

To date, few studies have empirically investigated the relationship between mitigation and adaptation in the public’s mind. Among these studies, two provide indirect evidence for a positive relationship: Farmers who believed that anthropogenic climate change was happening were more likely to endorse mitigation *and* adaptation measures than farmers who were sceptical about climate change [9]. It is, however, unclear to what extent these results apply to the general public. Yet a study conducted among homeowners—who may be more comparable to the general public—revealed similar findings: The more homeowners were aware of and concerned about climate change, the more money were they willing to pay for mitigation *and* adaptation measures [10]. Another study conducted with members of the general public found that the more people felt that climate change was a severe threat and the more they felt vulnerable, the more they were willing to mitigate [11]. However, severity and vulnerability did not predict adaptation, suggesting that people endorsed the two strategies for different reasons and that the relationship between mitigation and adaptation may be unsystematic. Overall, previous studies provide some evidence that specific groups (i.e., farmers and homeowners) endorse mitigation and adaptation strategies for similar reasons. However, very little is known about how the general public relates these two strategies to each other.

Against this backdrop, the aim of the present research is to further explore the relationship between endorsements of mitigation and adaptation by examining the correlations between different forms of mitigation and adaptation and by investigating people’s motives to mitigate and to adapt. To this end, we draw on a range of specific motivational variables that have been identified in previous research on individual responses to climate change.

## Individual’s Motivation to Endorse Mitigation and Adaptation

### Climate change scepticism

One factor that is crucial for people’s *motivation to mitigate* climate change is the extent to which people believe or doubt that climate change is real and will have serious effects. If people are sceptical about climate change, it is unlikely that they will act upon it. In line with this reasoning, some studies showed that higher levels of scepticism are associated with lower levels of endorsement of mitigation [9,12]. Others, however, have found no relationship between scepticism and mitigation [13,14].

There is some evidence that people low in scepticism are more likely to take actions to *adapt* to climate change than those high in scepticism [9,15]. However, it is also plausible that people who are sceptical about the reality and relevance of climate change may endorse adaptation measures. More specifically, if adaptation is understood as a possible future alternative to costly and unnecessary (from a sceptical point of view) mitigation efforts in the present, endorsing adaptation is an excuse for not taking any mitigation actions now [16–18] (see also [19]). In other words, it is possible that climate change sceptics favour adaptation over mitigation because it allows them to stall and block mitigation efforts.

### Attitudes

Until recently, climate change has almost exclusively been framed as an environmental problem [20]. Correspondingly, the general population is well aware that climate change will harm the environment [21] and their attitude towards nature and its protection should play some role in accounting for individual differences in mitigating climate change. Previous research confirms that people with strong pro-environmental attitudes are more likely to mitigate climate change [22,23]. Given that people who positively evaluate environmental protection usually also hold positive attitudes towards nature in general [24], we expect the relationship between attitude towards nature and endorsement of mitigation to be positive too.

Because adaptation has multiple foci—for example, maintaining human safety—the specific goal of protecting the environment is less prominent for this action than for mitigation [2]. Indeed, adaptive responses can, sometimes, entail actively changing the environment (e.g., construction of sea dikes to protect inhabited areas from future sea-level rise). We therefore assume that attitude towards nature and attitude towards environmental protection will be positively related to endorsement of adaptation, but not as strongly as these attitudes are related to endorsement of mitigation.

### Risk perceptions

Another important requisite for acting on a threat is the extent to which people feel at risk. Risks can be processed in two different ways: Analytically or affectively. The *analytical* processing mode is thought to be slow and deliberative. It deals with abstract symbols and numbers and is used to think about the future or hypothetical events. The *affective* processing mode, on the other hand, is fast and does not need any conscious effort. It relies on intuition, experiences, images, and associations [25]. Importantly, the two processing modes are not independent of each other. They work in parallel and influence our behaviour in conjunction with our decisions [26].

When used to explain people’s endorsement of *mitigation*, both analytic and affective appraisals of risks have been found to be reliable predictors: The more people considered climate change as a risk and the more negative feelings they had about it, the more they were willing to mitigate [12,13,22,23]. The relevance of risk perceptions for endorsement of *adaptation* measures is less clear [27]. Most studies that investigated risk perceptions explicitly related to climate change found no relevant (or only a negligible) link with people’s level of adaptation support [28,29]. This is unexpected, as it conflicts with theoretical work that considers risk perceptions as a crucial factor for people’s motivation to act in response to climate change [30] and is inconsistent with more general research on hazard preparedness, which typically finds that risk perceptions are strongly related to people’s willingness to take preventive measures [31].

Within analytical risk perception another differentiation should be made when dealing with climate change: that of generalised risks (e.g., to other people) versus the risks that are seen to be self-relevant to the individual contemplating action. Typically, people estimate the risks to others to be greater than those to themselves [32], something that can interfere with taking action because it deflects personal relevance and responsibility. This type of thinking has also been highlighted in relation to the risks of climate change: People generally perceive climate change as a distant threat, something that affects strangers, and that happens in remote times and places rather than here and now [12,33]. The perception of climate change as a distant threat could lead to the perspective that climate change risks are irrelevant to the individual and that there is no need for personal action. Put the other way around, when climate change is perceived as a phenomenon with proximal consequences, then people should also have a greater sense of urgency and responsibility, and should ultimately be more motivated to respond to climate change [34–36]. The assumption that proximal risk perceptions should increase people’s willingness to act seems especially relevant with regard to adaptation, given its predominantly local focus [6,37]. Haden and colleagues [38] provide some empirical evidence for this assumption. They found that the more farmers were concerned about future *local* water availability, the more they were willing to adapt to climate change by adopting new irrigation practices.

The distinction between proximal and distant risk perceptions also warrants some comments with regard to mitigation. One the one hand, the common assumption that an increased sense of proximal risks motivates people to mitigate [34,35] makes intuitive sense. On the other hand, the global scale at which mitigation works [4] suggests that distant risk perceptions could also be positively linked to endorsement of mitigation, especially among people who are *more* concerned about places at a more distant level than they are about proximal places [7]. Consistent with this reasoning, one study found that the more farmers were concerned about distant climate change, the more they were willing to adopt new mitigation practices [38]. Thus, there are theoretical reasons for and against the superiority of proximal risk perceptions over distant risk perceptions as a predictor of mitigation support; moreover, some evidence suggests that distant risk perceptions might be more relevant for motivating mitigation than proximal risk perceptions.

## Summary and Research Aims

An effective response to climate change requires the public to jointly endorse mitigation and adaptation measures. A key problem with this requirement is that very little research is available on how the general public relates these two strategies to each other. The main goal of this research was to assess the general public’s endorsement of different forms of mitigation and adaptation and to relate the levels of endorsement of these measures with each other. To this end we conducted online surveys in two European countries in which we (1) investigated correlations between different forms of endorsement of mitigation and adaptation and (2) compared how well social psychological predictor variables accounted for individual differences in endorsement of mitigation and adaptation. This second, more indirect approach of using social psychological variables as predictors of mitigation and adaptation enabled us to learn more about how (dis-)similar the reasons for endorsing the two response strategies were.

## Methods

### Design and Participants

In Spring 2010 we conducted one online survey in the UK and one in Switzerland. (The two studies were not originally planned and carried out with the intention of comparing them. This is also the reason why some questions and measures differ between the Swiss survey and the UK survey). In the UK sample, we recruited participants primarily through advertisements that were displayed on the websites of two newspapers with an assumed different readership (“The Daily Mirror” and “The Independent”). Participants were told that the survey was about current affairs and how they used different media. Participants were then instructed on how to fill in the questionnaire and about their rights. Informed consent was attained by asking participants to only continue if they had read and understood the provided instructions and information and if they were willing to participate in our survey. Ethical approval for this study was granted by the ethics committee of the Psychology Department, University of Exeter; the consent procedure is common practice for online studies and was not explicitly appraised by the ethics committee

After some filler questions participants were assigned an allegedly random topic of current interest—which was always “climate change”. The survey asked participants about their views on climate change and concluded with socio-demographic questions (S2 and S3 Tables). The proportion of females among the 612 participants was 41.5%, and the proportion of males was 45.8% (12.7% did not report their sex). On average, people were 39.3 years old (*SD* = 13.31; range: 16 to 83).

In the Swiss survey, we sent an email to people who had participated in a previous research project on environmental attitudes and pro-environmental behaviour [24] and asked them to take our online survey. The survey was framed as a follow-up to examine how attitudes and behaviours develop over time. The first part of the questionnaire asked participants about their attitudes towards nature and environmental protection. Next, the survey asked about their views on climate change and concluded with basic demographic questions. Of the 316 participants, seven were excluded because they lived in a country other than Switzerland. Of the final sample, 48.5% were females and 51.5% were males; the sample's mean age was 36.6 years (SD = 15.2; range: 19 to 81). The data were collected under the affiliation of the University of Zürich. At the time of our study, the University of Zürich had no standing committee on ethics and no institutional review board required approval for research in the social sciences. We, however, certify that this research adheres to the ethical guidelines for research with human participants of the American Psychological Association [39]. Informed consent was obtained in the same way as in the UK Study.

### Measures

#### Predictor variables

To assess the degree to which people were *sceptical* about human-made climate change, we adopted seven previously used items [21,40] and added an item that referred to the previous unusually cold winter. Participants were asked to indicate how much they agreed with these statements (see S1 Table for all items used). The seven items formed a reliable scale (Cronbach’s α in the UK survey =. 85, Cronbach’s α in the Swiss survey =. 87).

To assess *attitude towards environmental protection* (Swiss sample), we used a behaviour-based attitude measure with 50 items [41]. This instrument included 18 items with a yes/no response and 32 items with a frequency scale. The 50 items of the environmental attitude measure formed a reliable scale (α =. 86).

The more general *attitude towards nature* consisted of 40 items (Swiss sample), of which 26 were presented as behavioural self-reports and 14 as evaluative statements [24]. The 40 items formed a reliable scale (α =. 84).

In the UK survey we used four items to assess people’s *attitude towards addressing climate change* (items taken from [21]). The reliability of this scale was acceptable (α =. 59).

To assess *risk perceptions*, we asked people to judge the likelihood that seven (identical) risks [12,22,23] would occur as a consequence of climate change either close to or far away from where they live. The seven items formed reliable scales both for the proximal (α_UK_ =. 83; α_Switzerland_ =. 76) and distant level (α_UK_ =. 90; α_Switzerland_ =. 83).

In the Swiss survey, we assessed *affective risk perception* by asking participants about their emotional reaction when they thought about climate change (1 = very positive, 7 = very negative; [42]). In the UK survey, participants were first asked if they had any negative feelings about climate change (1 = *none*). If they replied with “yes”, they were also asked to rate the degree of their negative feelings (2 = *slightly*, 5 = *extremely*).

#### Dependent measures

To measure participants’ *support for mitigation policies*, respondents were presented with a selection of steps to decrease the amount of greenhouse gases “as a society” and then asked how they would vote on them in a national referendum [23]. We adapted eight questions—some with slightly different wordings—from the literature [43,44] and created six new questions. The 14 propositions to mitigate climate change as a collective formed a reliable scale (α_UK_ =. 90, α_Switzerland_ =. 89).

Participants were presented with a brief introduction (see S1 Fig) explaining the need and the rationale underlying the idea of adaptation. To assess *support for pro-active adaptation policies*, we developed a catalogue of 15 adaptation measures that were guided by adaptation research [45] and by strategies from large cities [46]. The proposed adaptation measures focused on conservation of species, protection against water scarcity, heat, and floods. Participants were told that there were many steps that “we can take as a society to adapt to climate change” and then asked to indicate how much they would support different adaptation measures. The 15 items formed a reliable scale (α_UK_ =. 89, α_Switzerland_ =. 83).

We used ten items to assess *people’s future intentions to engage in behaviours to mitigate* climate change (UK sample) and asked them how likely they were to take these actions. The topics revolved around mobility, energy saving, consumption, and political behaviours. The proposed ten actions formed a reliable scale in the UK survey (α =. 78). For lack of space we could not include this measure in the Swiss survey.

We also presented (UK) participants with eight actions they could individually take to adapt to climate change impacts. The eight personal behavioural intentions to adapt formed a reliable scale (α =. 81). (Again, there was no space for these questions in the Swiss survey).

To explore whether our newly developed dependent variables measure one-dimensional concepts, we conducted a series of exploratory factor analyses. We first ran two principal axis factoring analyses that included either the personal behavioural intentions to mitigate or to adapt (since personal behavioural intentions were not assessed in Switzerland, the analyses were only performed with the UK sample). We then explored (in both samples) the dimensionality of the mitigation and adaptation policy support items. All six analyses revealed that the items were suitable for factor analysis (KMO and Bartlett’s test, S4–S7 Tables). Although the presence of more than one factor with an eigenvalue larger than one would suggest keeping several factors according to the Kaiser criterion [47], the eigenvalues of the first factor were considerably larger than the ones of the second factor (scree plots, S2 Fig). We therefore decided to retain the anticipated and theoretically more meaningful one-dimensional solutions.

## Results

### Direct relationships between mitigation and adaptation

The Pearson correlations in Table 1 provide direct information for how people’s willingness to endorse mitigation and adaptation—either on a personal or societal level—are related. Most importantly, all correlation coefficients between mitigation and adaptation are positively related with medium to large effect sizes (.44 ≤ *r* ≤. 66). The strongest relationships were found between variables that were on the same level of implementation (either personal or societal): The correlation between support for mitigation and adaptation policies was *r* =. 66 in the UK and *r* =. 61 in Switzerland. People’s personal behavioural intentions to mitigate climate change and adapt to its consequences were also strongly correlated *r* =. 60.

**Table 1:** Descriptive statistics and bivariate correlations between predictor variables and dependent variables.

	M_UK_ / M_CH_	SD_UK_ / SD_CH_	2	3	4	5	6	7	8	9
1 Attitude towards nature	/ 3.39	/ 0.58	.47***	-.11	.07	.30***	.26***	.33***	.27***	
2 Attitude towards addressing climate change (UK) / environmental protection (Switzerland)	3.26 / 3.62	0.70 / 0.50		-.36***	.19**	.25***	.36***	.60***	.35***	
3 Scepticism	2.45 / 2.04	0.82 / 0.75	-.67***		-.41***	-.32***	-.58***	-.61***	-.41***	
4 Affective risk perception	2.50 / 5.49	1.42 / 1.08	.49***	-.52***		.18**	.41***	.31***	.22***	
5 Local risk perception	3.10 / 2.85	0.72 / 0.63	.44***	-.41***	.39***		.61***	.43***	.36***	
6 Global risk perception	3.87 / 3.97	0.74 / 0.62	.53***	-.56***	.44***	.68***		.61***	.54***	
7 Mitigation policy support	3.35 / 3.66	0.77 / 0.74	.55***	-.56***	.43***	.45***	.53***		.61***	
8 Adaptation policy support	3.69 / 3.48	0.62 / 0.54	.43***	-.44***	.34***	.36***	.48***	.66***		
9 Mitigation personal intentions	3.44 / -	0.67 / -	.42***	-.41***	.36***	.40***	.44***	.60***	.47***	
10 Adaptation personal intentions	2.88 / -	0.78 / -	.36***	-.33***	.29***	.38***	.30***	.44***	.42***	.61***

*Note*. To the right of the means (M) and standard deviations (SD), the figures below the diagonal refer to the United Kingdom (UK) survey, those above to the Swiss (CH) survey. As we did not assess personal intentions in the Swiss survey, these cells do not contain any figures.

*** Stands for *p* < .001

** stands for *p* < .01

* stands for *p* < .05

Taken together, the correlation coefficients show that when a person endorses any form of mitigation—for example introducing new taxes—she is likely to also endorse any other way of mitigating climate change—for example by using public transport more often—and to endorse measures to adapt to impacts of climate change (i.e., positive relationship). Of course, this also means that endorsement of any adaptation measure also entails endorsement of mitigation measures. This speaks against the idea that people think of mitigation and adaptation as alternative or mutually exclusive strategies to respond to climate change.

### Indirect information about the relationship of public endorsement of mitigation and adaptation

We built hierarchical regression equations with four different blocks to predict policy support and personal behavioural intentions in multiple regressions. The first block included the level of individual scepticism about climate change and either an attitude that specifically related to addressing climate change (UK) or two more general attitudes related to environmental protection and nature (Switzerland). The second block consisted of the affective risk perception and the third block included analytical risk perceptions, which were divided into proximal and distant risks. These three models were instructive because they allowed us to evaluate the predictive power of each set of predictors and to learn more about how well these specific predictors can account for the variance in the dependent variable in relation to each other (e.g., when the focus was to compare proximal vs. distant risk perceptions).

To account for a potential overlap between the predictor variables, we also ran models in which we included all predictors simultaneously. These full models allowed us to assess the predictive power of each variable while all other predictors were held constant and to identify the predictors that best explain the variance in the dependent variable.

#### Support for mitigation and adaptation policies

Attitudes and sceptical beliefs (Model 1) did the best job of explaining people’s *support for mitigation policies* (*R* ^*2*^_*adjusted*_ =. 37 /. 54, Table 2): The more positive people’s attitude towards nature and the stronger their attitude to protect the environment (Switzerland) or to address climate change (UK) and the less they were sceptical about climate change, the more they were willing to support mitigation policies. (Analytical) risk perceptions (proximal and distant risks) represented the second most powerful predictor of mitigation support—as can be seen in the amount of variance explained as a separate predictor block (*R* ^*2*^_*adjusted*_ =. 29 /. 37, Model 3, Table 2) and the size of their Beta-weights in the Full Models (Table 2).

**Table 2:** Results of multiple regression analyses predicting policy support in the surveys conducted in the UK and in Switzerland.

	Mitigation policy support																Adaptation policy support															
	1				2				3				Full				1				2				3				Full			
Predictors	*B*	*SE*	*β*	*Sig*.	*B*	*SE*	*β*	*Sig*.	*B*	*SE*	*β*	*Sig*.	*B*	*SE*	*β*	*Sig*.	*B*	*SE*	*β*	*Sig*.	*B*	*SE*	*β*	*Sig*.	*B*	*SE*	*β*	*Sig*.	*B*	*SE*	*β*	*Sig*.
*UK survey*																																
Attitude towards addressing climate change	.35	.05	.32	***									.24	.05	.22	***	.21	.05	.23	***									.11	.05	.13	*
Scepticism	-.33	.04	-.35	***									-.23	.05	-.24	***	-.22	.04	-.29	***									-.12	.04	-.16	**
Affective risk					.23	.02	.43	***					.04	.02	.08						.15	.02	.34	***					.03	.02	.06	
Proximal risk									.18	.05	.17	***	.12	.05	.11	*									.05	.05	.06		.02	.05	.02	
Distant risk									.43	.05	.41	***	.17	.06	.16	**									.37	.04	.44	***	.24	.05	.28	***
Adjusted *R* ^*2*^ */ N*			.37	532			.18	513			.29	568			.42	485			.22	527			.12	491			.23	540			.28	485
*Swiss survey*																																
Attitude towards nature	.12	.06	.10	*									.05	.07	.04		.15	.05	.17	**									.10	.06	.10	
Attitude towards environmental protection	.57	.07	.39	***									.53	.08	.36	***	.16	.07	.15	*									.12	.08	.11	
Scepticism	-.45	.04	-.46	***									-.31	.06	-.31	***	-.25	.04	-.34	***									-.10	.06	-.14	
Affective risk					.21	.04	.31	***					-.00	.03	-.00						.11	.03	.22	***					-.01	.03	-.03	
Proximal risk									.10	.07	.09		.08	.07	.07										.04	.05	.05		.02	.06	.02	
Distant risk									.66	.07	.56	***	.29	.08	.25	***									.44	.05	.51	***	.34	.08	.39	***
Adjusted *R* ^*2*^ */ N*			.54	307			.09	215			.37	309			.59	213			.23	307			.05	215			.28	309			.32	213

*Note*. *B* = Unstandardized regression coefficient, *SE* = Standard Error, *β* = Standardized regression coefficient, *Sig*. = level of statistical significance.

*** stands for *p* <. 001

** stands for *p* <. 01

* stands for *p* <. 05

When the potential overlap between the predictors was taken into account in the Full Models (Table 2), scepticism and attitude towards addressing climate change (UK survey) and attitude towards environmental protection (Swiss survey) independently explained the largest proportion of variance of support for mitigation policies, followed by distant risk perceptions. Particularly interesting is the predictive power of people’s attitude towards environmental protection, which independently explained 13% of the variance in mitigation policy support (Table 2). This suggests that for many people protecting the environment in general and supporting mitigation policies go hand in hand.

With regard to *support for adaptation policies*, the attitude and sceptical beliefs block (*R* ^*2*^_*adjusted*_ =. 22 /. 23, Model 1, Table 2) explained slightly less of the variance than the block containing risk perceptions (*R* ^*2*^_*adjusted*_ =. 23 /. 28, Model 3). In the Full Model the single most powerful predictor of support for adaptation policies is *distant* risk perception: The more likely people judge climate risks to affect remote places, the more they are willing to support adaptation policies (Table 2). The role of proximal risk perceptions as a predictor of support for adaptation policies, however, was negligible.

#### Personal behavioural intentions to mitigate and to adapt

The model containing attitudes and sceptical beliefs (Model 1, Table 3) and the one featuring analytical risk perceptions (Model 3, Table 3) both explained 20% of the variance in people’s *personal behavioural intentions to mitigate*. When directly compared, attitude towards addressing climate change and sceptical beliefs were equally strong predictors of mitigation intentions (Model 1, Table 3). In the Full Model, however, the contribution of scepticism to explaining personal behavioural intentions to mitigate was no longer statistically significant. At first glance, this stands in contrast to the important role sceptical beliefs played as a predictor of mitigation policy support. Yet the inferiority of the scepticism measure with regard to the statistically significant predictors is small in terms of the size of the Beta-weights (*Δβ* ≤. 06).

**Table 3:** Results of multiple regression analyses predicting personal intentions in the survey conducted in the UK.

	Mitigation intentions																Adaptation intentions															
	1				2				3				Full				1				2				3				Full			
Predictors	*B*	*SE*	*β*	*Sig*.	*B*	*SE*	*β*	*Sig*.	*B*	*SE*	*β*	*Sig*.	*B*	*SE*	*β*	*Sig*.	*B*	*SE*	*β*	*Sig*.	*B*	*SE*	*β*	*Sig*.	*B*	*SE*	*β*	*Sig*.	*B*	*SE*	*β*	*Sig*.
Attitude towards addressing climate change	.25	.05	.27	***									.14	.05	.15	**	.27	.06	.24	***									.17	.07	.16	**
Scepticism	-.19	.04	-.23	***									-.09	.05	-.11		-.16	.05	-.17	**									-.11	.06	-.11	
Affective risk					.17	.02	.36	***					.05	.02	.10	*					.16	.02	.29	***					.04	.03	.07	
Proximal risk									.17	.05	.18	***	.13	.05	.14	*									.36	.06	.33	***	.32	.06	.29	***
Distant risk									.28	.05	.31	***	.14	.06	.15	*									.08	.06	.08		-.08	.07	-.07	
Adjusted *R* ^*2*^ */ N*			.20	499			.13	502			.21	554			.26	485			.14	496			.08	497			.15	548			.19	485

*Note*. *B* = Unstandardized regression coefficient, *SE* = Standard Error, *β* = Standardized regression coefficient, *Sig*. = level of statistical significance.

*** stands for *p* <. 001

** stands for *p* <. 01

* stands for *p* <. 05

In contrast to support for mitigation policy, the superiority of distant risk perceptions as a predictor of personal behavioural intentions to mitigate shrunk in direct comparison to proximal risk perceptions (Model 3, Table 3) and completely disappeared when the remaining predictors were added (Full Model, Table 3). In this Full model, all predictors—with the exception of scepticism—made a statistically significant and a similarly large contribution to explain people’s intention to take personal mitigation actions.

When combined in Model 1, attitude towards addressing climate change and scepticism (*R* ^*2*^_*adjusted*_ =. 14, Table 3) and analytical risk perceptions (*R* ^*2*^_*adjusted*_ =. 15, Model 3, Table 3) equally well predicted *personal behavioural intentions to adapt*. In the Full Model, proximal risk perceptions outperformed the other predictors: The perception of (more) *proximal* risks was by far the best predictor of people’s willingness to take personal adaptation actions (Table 3).

The other variables that achieved statistical significance as predictors of personal behavioural intentions to adapt in the Full Model were the degree of people’s scepticism and their attitudes towards addressing climate change. The less sceptical people were about climate change and the more positively their attitudes towards addressing climate change, the more they were ready to take personal adaption actions.

## Discussion, Limitations, and Conclusions

### Discussion

This study provides consistent evidence from two countries that public endorsement of mitigation and adaptation are strongly linked to each other and that the two response strategies are endorsed for similar reasons. People who were willing to mitigate climate change—be it on a personal level by changing their behaviour or on a societal level by supporting policies—also supported personal and societal steps to adapt to negative consequences from climate change, and vice versa.

It also seems that the psychological processes involved in peoples’ motivation to respond to climate change are very similar for these two strategies: People who believe that climate change is real, who have positive attitudes about protecting the environment and about addressing climate change, and who perceive climate change as a risk, are willing to endorse both mitigation and adaptation strategies.

The finding that surprised us most, and that opens new avenues for future research, concerns the relationship between risk perceptions and the level at which response strategies are implemented. A common idea is that people are not willing to address climate change because they perceive it as a distant threat and that their motivation could be increased by making local consequences more salient [34,35]. If “localising” is an effective means to increase individual engagement with climate change, as is often expected, then proximal risk perceptions should outperform distant risk perceptions as a predictor for every dependent variable. However, it was *distant* risk perceptions that better predicted people’s willingness to support mitigation and adaptation policies rather than *proximal* risk perceptions (Table 2). Neither was proximal risk perception superior in terms of personal behavioural intentions to mitigate (Table 3); both proximal and distant risk perceptions uniquely contributed to the prediction of personal behavioural intentions. It was only with regard to personal adaptation intentions that we found the expected superiority of proximal risk perceptions as a predictor.

Construal Level Theory [48], a dominant theory in the current psychological literature, provides a possible explanation for the unexpected associations between proximal risks and personal behavioural intentions as well as between distant risks and policy support. Construal Level Theory posits that human perceptions of the world and the way individuals make judgments depend on the psychological distance between the self and the events or objects being dealt with. Low psychological distance means that we are focused on ourselves, our current geographic location, and the present time. Conversely, when psychological distance is high, then we focus on other people, faraway places and the distant future or past.

The variables we used to assess analytical risks and willingness to respond to climate change neatly map onto two dimensions of psychological distance: Proximal and distant risk perceptions can be mapped onto the dimension of spatial distance; personal behavioural intentions and policy support can be mapped onto the social dimension of psychological distance (*I* vs. *we* do something). Perhaps the unexpected strong relationships we found between proximal risk perceptions and personal behavioural intentions on the one hand, and between distant risk perceptions and policy support on the other hand, are due to spontaneous matches with regard to psychological distance. Proximal risk perceptions are psychologically *proximal on the spatial dimension* and personal behavioural intentions can be regarded as *proximal on the social dimension* (it is the individual who forms the intentions). Likewise, the *spatially remote* distant risk perceptions can be matched to support for policies, which can be regarded as *distant on the social dimension* (policies involve strangers and collective action). Importantly, the proposed spontaneous matches would be consistent with the empirically corroborated proposition of Construal Level Theory saying that different dimensions of psychological distance (e.g., social and spatial distance) are related to each other [48]. To some extent this proposition has also been observed with regard to people’s perception of climate change [36]. It is therefore plausible that the observed links between the risk perspective and level of implementation are due to a similar level of psychological distance from the individual and, therefore, due to a corresponding level of mental construal.

This Construal Level Theory interpretation of our findings resembles the proposition that mitigation and adaptation strategies are in themselves linked to specific construal levels. Haden and colleagues (2012) argued that because mitigation is a collective problem that requires global action (i.e., involving greater spatial and social distance), this strategy is represented more abstractly than adaptation, which instead deals with local problems that directly affect the individual (i.e., involving less spatial and social distance). Our findings add to this literature by showing that the distinction of personal versus societal responses to climate change may be helpful to understand how different aspects of psychological distance affect endorsement of mitigation and adaptation strategies.

The Construal Level Theory perspective leads to some counter-intuitive corollaries that are relevant for climate change communication. The relationships we observed between spatially varying risk perspectives and the two levels of implementation imply that appeals to change personal behaviour and to increase support for policies are most effective when they are combined with spatial information that is consistent with their respective construal. This logic is consistent with the reasoning of Construal Level Theory and with empirical findings on the effectiveness of matching construal levels [49]. The importance of matching levels could also explain why previous attempts to increase people’s engagement with climate change by portraying proximal impacts (i.e., low-level construal) were not successful in increasing support for policies and strengthening attitudes towards climate change mitigation (both high-level construals [50,51]). In short, on the basis of our results and current theory, we suggest that a proximal focus does not *per se* lead to more involvement and stronger motivation to act on climate change (for a similar argument, see [36]). Rather, climate change communication could be much more effective if different levels of construals are intentionally matched: Focussing people’s attention on local climate change seems well suited to enhance their willingness to take personal actions, probably more so if these actions are related to adaptation [38]. However, with regard to support for policies it seems more effective to address the issue of climate change on a bigger scale and therefore remove climate change spatially from people rather than bring it closer to them [51], especially if these policies concern mitigation [38].

### Limitations and directions for future research

At least three limitations might have affected the findings of this research. The first issue we would like to discuss is the small amount of variance affective risk perception could explain with regard to the dependent variables. However, we believe that the weak predictive power of affective risk does *not* indicate that this variable is irrelevant as a motivation to address climate change (see also the successful use of affect in previous studies [12,42]). Instead, we believe that this weakness reflects the potential for methodical improvement, such as including several items rather than a single-item question.

Second, our samples were not representative of either the UK or Switzerland. However, given the moderate size of the samples (*N* = 616 / 309) and given that the participants in the two samples cover a broad range of socio-demographic characteristics (S2 and S3 Tables), we are confident that our samples are diverse enough to study relationships between different social-psychological variables. We also think that given the different recruiting strategies and considering that survey contexts were markedly different, the highly similar results from the two samples speak of the external validity of our findings. However, without a more representative sample, we do not know whether our findings are limited to persons who are younger and comparatively better educated.

The third shortcoming of this research has to do with the selection of predictor variables. Although we included a variety of psychological processes to study similarities and differences between endorsement of mitigation and adaptation, there are other potentially relevant variables—for example, the extent to which people believe that the proposed measures will be effective (i.e., efficacy beliefs [28,30,52]) and see themselves as personally capable of responding to climate change (i.e., self-efficacy beliefs [30,35,52]. Other relevant processes might include perceived costs of responses [52], experience with the effects of climate change [35], or beliefs and opinion of relevant others [28]. Studying these alternative predictors of endorsement of mitigation and adaptation could reveal that the motives to mitigate and to adapt vary more strongly than suggested by our findings.

### Conclusions

In responding to climate change, mitigation and adaptation go hand in hand—not just in pleas from the scientific community but also in people’s minds. Yet, sometimes one hand is needed more than the other. The better the understanding of how to mobilise each hand to do specific things, for example by focussing on proximal risk perceptions to foster the willingness to take personal adaptation actions and by appealing to existing attitudes to increase support for mitigation policies, the more effective responses to the challenges of climate change will be.

## Supporting Information

S1 Data Raw data of the UK sample. (DAT)

S2 Data Raw data of the Swiss sample. (DAT)

S1 Fig Texts used to explain what adaptation to climate change is. (DOCX)

S2 Fig Scree plots from principal axis factor analyses. (EPS)

S1 Table Scales and items used in the UK and Swiss sample. (DOCX)

S2 Table Demographic characteristics of the sample collected in the United Kingdom. (DOCX)

S3 Table Demographic characteristics of the sample collected in Switzerland. (DOCX)

S4 Table Summary of personal mitigation intentions, factor loadings, and communalities from principal axis factor analysis. (DOCX)

S5 Table Summary of personal adaptation intentions, factor loadings, and communalities from principal axis factor analysis. (DOCX)

S6 Table Summary of mitigation policy support items, factor loadings, and communalities from principal axis factor analysis. (DOCX)

S7 Table Summary of adaptation policy support items, factor loadings, and communalities from principal axis factor analysis. (DOCX)
