# Supplementary material for: Hand in Hand: Public Endorsement of Climate Change Mitigation and Adaptation
Source: PLoS One. 2015 Apr 29;10(4):e0124843. doi: 10.1371/journal.pone.0124843 (PMC4414563; doi:10.1371/journal.pone.0124843)
Supplement: S1 Fig — (DOCX) [file pone.0124843.s003.docx]

Figure S1*.* Texts used to explain what adaptation to climate change is.

**UK Study:**

Scientists argue that due to past emissions of greenhouse gases the planet is already committed to a certain amount of climate change over the next couple of decades. This makes adaptation to the positive and negative impacts of climate change unavoidable. There are many steps we can take as a society to adapt to climate change.

**Swiss Study (translated):**

Adaptation to climate change refers to measures that support natural and human systems (e.g., agriculture) to suffer as little as possible from the consequences of climate change. Scientists argue that because of past greenhouse gas emissions a certain extent of climate change is already expected within the next decades. It is therefore unavoidable to adapt to the negative (and positive) consequences of climate change. There are many possibilities of how we as a society can adapt to climate change.
