# Supplementary material for: Hand in Hand: Public Endorsement of Climate Change Mitigation and Adaptation
Source: PLoS One. 2015 Apr 29;10(4):e0124843. doi: 10.1371/journal.pone.0124843 (PMC4414563; doi:10.1371/journal.pone.0124843)
Supplement: S1 Table — (DOCX) [file pone.0124843.s005.docx]

| *S1 Table.* Scales and items used in the UK and Swiss sample. |  |  |
| --- | --- | --- |
| Scales, items, and response formats | UK sample | Swiss sample |
| *Proximal risk perceptions (*N *= 7),* 1 = *very unlikely,* 5 = *very likely* |  |  |
| Water shortages will occur where I live | X | X |
| Starvation and food shortages will occur where I live | X | X |
| Standard of living of many people in the UK / Switzerland will decrease | X | X |
| Health problems in the UK / Switzerland will increase | X | X |
| Number of species lost in the UK / Switzerland will increase | X | X |
| The UK's / Switzerland’s economic situation will deteriorate | X | X |
| More flooding will occur where I live | X | X |
| *Distant risk perception (*N *= 7) ,* 1 = *very unlikely,* 5 = *very likely* | X | X |
| Worldwide water shortages will occur | X | X |
| Starvation and food shortages will occur in much of the world | X | X |
| Standard of living of many people in the world will decrease | X | X |
| Health problems in the world will increase | X | X |
| Number of species lost in the world will increase | X | X |
| The worlds economic situation will deteriorate | X | X |
| More flooding will occur worldwide | X | X |
| *Mitigation policy support (*N *= 14),* 1 = *definitely no,* 5 = *definitely yes* |  |  |
| Ban the driving of cars in certain areas | X | X |
| Ban the production of vehicles with gas / fuel mileage below 75 miles per gallon (very fuel efficient) | X | X |
| Increased fuel and diesel taxes | X | X |
| Increased household electricity taxes | X | X |
| Congestion charging on busy roads | X | X |
| Air travel taxation (e.g. on ticket prices) | X | X |
| Subsidies for electric (emission-free) vehicles | X | X |
| Subsidies for house insulation | X | X |
| Information campaigns about negative climate effects caused by car and aeroplane travel | X | X |
| Introducing labels stating carbon content | X | X |
| Teach children about the causes, consequences, and potential solutions to climate change | X | X |
| Subsidies for the household production of green energy (e.g., small wind turbines and solar panels) | X | X |
| Tax for the protection of tropical rain forests | X | X |
| Increasing general taxation to pay for public transport | X | X |
| Table S1 continues |  |  |

| Table S1 continued |  |  |
| --- | --- | --- |
| Scales and items | UK sample | Swiss sample |
| *Adaptation policy support (*N *= 15),* 1 = *definitely no,* 5 = *definitely yes* |  |  |
| New spatial planning to reduce the risk of flooding | X | X |
| Obligatory integration of climate risk and adaptation assessments into business planning | X | X |
| Train Health Services staff to identify and advise on heat stress risks | X | X |
| Upgrade all flood defences to a higher standard | X | X |
| Protection and creation of wetlands (improves flood protection and contributes to biodiversity) | X | X |
| Requirement to fit houses with water resistant door and window frames in flood risk areas | X | X |
| Close access to vulnerable places, including some recreation areas, marinas, and hiking trails | X | X |
| Reduce pressure on systems or areas at risk (e.g., fewer fishing and hunting licences) | X | X |
| Relocation of dwellings away from flood-prone areas | X | X |
| Increase national development assistance to help developing countries to adapt to climate change | X | X |
| Produce and distribute guidance on how to avoid heat stress | X | X |
| Hosepipe restrictions during the summer | X | X |
| Introduce building codes to make houses more thermally comfortable with longer and hotter summers | X | X |
| Tax to establish a fund to alleviate unavoidable climate change impacts in the UK / in Switzerland | X | X |
| Creation of habitat corridors for animals (e.g., bridges over motorways) | X | X |
| *Mitigation intentions (*N *= 10), 1 = very unlikely, 5 = very likely* |  |  |
| Choose a car that gets good fuel mileage | X |  |
| Install (more) insulation at home | X |  |
| Car sharing / Use car sharing pools | X |  |
| Using public transport (more often) | X |  |
| Walking and cycling (more) | X |  |
| Replace older appliances with more energy efficient new models (e.g., refrigerators) | X |  |
| Join an environmental group | X |  |
| Carbon offset flights | X |  |
| Eat less meat | X |  |
| Reduce the number of new things you buy | X |  |
| Table S1 continues |  |  |

| Table S1 continued |  |  |
| --- | --- | --- |
| Scales and items | UK sample | Swiss sample |
| *Adaptation intentions (*N *= 8),* *1 = very unlikely, 5 = very likely* |  |  |
| Repaint your (future) house in a lighter colour (less heat absorption in the summer) | X |  |
| Buy flood insurance for your (future) house | X |  |
| Install a water re-use system at home (avoid water shortages during droughts) | X |  |
| Donate money to preserve species at risk from climate change | X |  |
| Persuade relatives or friends to move away from flood plains | X |  |
| Fit water saving device in your cistern to save when flushing | X |  |
| Read about how to avoid heat stress during heat waves | X |  |
| Donate money for settlement relocation projects in developing countries | X |  |
| *Scepticism (*N *= 7),* 1 = *strongly disagree,* 5 = *strongly agree* |  |  |
| Climate change is caused only by natural processes | X | X |
| Experts are agreed that climate change is a real problem (reverse coded) | X | X |
| The media is often too alarmist about issues to do with climate change | X | X |
| The evidence for climate change is unreliable | X | X |
| I am uncertain if climate change is happening | X | X |
| There’s no such thing as climate change | X | X |
| The cold and snowy winter was a proof that climate change is not happening | X | X |
| *Attitude addressing climate change (*N *= 4),* 1 = *strongly disagree,* 5 = *strongly agree* |  |  |
| I do not mind if it gets a bit warmer in the UK | X |  |
| New technologies can solve global warming, without individuals having to make big changes in their lives | X |  |
| I feel a moral duty to do something about climate change (reverse coded) | X |  |
| Radical changes to society are needed to tackle climate change (reverse coded) | X |  |
| *Attitude towards environmental protection* |  |  |
| 50 items, see Kaiser and Wilson (2004) |  | X |
| *Attitude towards nature* |  |  |
| 40 items, see Brügger et al. (2011) |  | X |
| *Note.* X = item used in the respective sample. |  |  |
