# Supplementary material for: Hand in Hand: Public Endorsement of Climate Change Mitigation and Adaptation
Source: PLoS One. 2015 Apr 29;10(4):e0124843. doi: 10.1371/journal.pone.0124843 (PMC4414563; doi:10.1371/journal.pone.0124843)
Supplement: S2 Table — (DOCX) [file pone.0124843.s006.docx]

*S2 Table.* Demographic characteristics of the sample collected in the United Kingdom.

|  | % |
| --- | --- |
| Gender |  |
| Female | 47.6 |
| Male | 52.4 |
| Age |  |
| 16–24 | 16.4 |
| 25–44 | 46.4 |
| 45–64 | 34.6 |
| 65 and over | 2.6 |
| Household yearly income |  |
| Up to £7000 | 7.0 |
| £7001 - £14000 | 6.8 |
| £14001 - £21000 | 9.6 |
| £21001 - £28000 | 12.1 |
| £28001 - £34000 | 7.0 |
| £34001 - £41000 | 11.8 |
| £41001 - £48000 | 11.2 |
| £48001 - £55000 | 7.9 |
| £55001 - £62000 | 4.6 |
| £62001 or more | 10.1 |
| Prefer not to say | 11.8 |
| Qualifications |  |
| O-levels | 13.1 |
| A-level or equivalent | 21.2 |
| Higher national diploma | 10.4 |
| Degree or equivalent | 33.0 |
| Post-graduate qualification | 22.2 |
| Area density |  |
| Remote Area | 5.2 |
| Village | 18.3 |
| Town | 29.4 |
| City (Suburban) | 27.0 |
| City (Central/Inner Area) | 20.0 |
| Preferred political party |  |
| Labour | 27.1 |
| Conservative | 20.2 |
| Liberal Democrats | 40.2 |
| Green | 12.5 |
