# Supplementary material for: Hand in Hand: Public Endorsement of Climate Change Mitigation and Adaptation
Source: PLoS One. 2015 Apr 29;10(4):e0124843. doi: 10.1371/journal.pone.0124843 (PMC4414563; doi:10.1371/journal.pone.0124843)
Supplement: S3 Table — (DOCX) [file pone.0124843.s007.docx]

*S3 Table.* Demographic characteristics of the sample collected in Switzerland.

|  | % |
| --- | --- |
| Gender |  |
| Female | 48.5 |
| Male | 51.5 |
| Age |  |
| 16–24 | 17.5 |
| 25–44 | 55.2 |
| 45–64 | 17.6 |
| 65 and over | 9.7 |
| Do you have a university degree? |  |
| No | 35.5 |
| Yes | 64.5 |
| Which of the following best describes what you are currently doing? |  |
| I study | 46.1 |
| I work | 35.5 |
| I run a household | 1.4 |
| I run a household with kids | 3.7 |
| I am retired | 7.8 |
| Something else | 5.4 |
| Area density |  |
| Population < 4,000 | 23.5 |
| 4,000–10,000 | 20.4 |
| 10,000–50,000 | 22.6 |
| 50,000–100,000 | 7.4 |
| Population > 100,000 | 26.1 |

*Note.* With the exception of age, the socio-demographic characteristics of this sample were assessed two years before this study [24]. Some figures might therefore be out-dated.
