# Supplementary material for: Hand in Hand: Public Endorsement of Climate Change Mitigation and Adaptation
Source: PLoS One. 2015 Apr 29;10(4):e0124843. doi: 10.1371/journal.pone.0124843 (PMC4414563; doi:10.1371/journal.pone.0124843)
Supplement: S4 Table — (DOCX) [file pone.0124843.s008.docx]

*S4 Table.* Summary of personal mitigation intentions, factor loadings, and communalities from principal axis factor analysis.

|  | UK sample | | |
| --- | --- | --- | --- |
| Item | Factor loading |  | *h^2^* |
| Join an environmental group | .59 |  | .35 |
| Carbon offset flights | .58 |  | .33 |
| Reduce the number of new things you buy | .57 |  | .33 |
| Walking and cycling (more) | .55 |  | .30 |
| Using public transport (more often) | .52 |  | .27 |
| Eat less meat | .51 |  | .26 |
| Choose a car that gets good gas mileage | .51 |  | .26 |
| Replace older appliances with more energy efficient new models (e.g., refrigerators) | .49 |  | .24 |
| Car sharing | .46 |  | .21 |
| Install more insulation at home | .45 |  | .20 |
| Kaiser-Meyer-Olkin measure of sampling adequacy | .83 |  |  |
| Bartlett's test of sphericity | *p* < .001 |  |  |
| Eigenvalue | 3.47 |  |  |
| % of variance | 34.66 |  |  |

*Notes. h^2^* = communality.
