# Supplementary material for: Hand in Hand: Public Endorsement of Climate Change Mitigation and Adaptation
Source: PLoS One. 2015 Apr 29;10(4):e0124843. doi: 10.1371/journal.pone.0124843 (PMC4414563; doi:10.1371/journal.pone.0124843)
Supplement: S5 Table — (DOCX) [file pone.0124843.s009.docx]

*S5 Table.* Summary of personal adaptation intentions, factor loadings, and communalities from principal axis factor analysis.

|  | UK sample | | |
| --- | --- | --- | --- |
| Item | Factor loading |  | *h^2^* |
| Persuade relatives or friends to move away from flood plains | .74 |  | .55 |
| Install a water re-use system at home (avoid water shortages during droughts) | .68 |  | .47 |
| Donate money for settlement relocation projects in developing countries | .60 |  | .35 |
| Donate money to preserve species at risk from climate change | .59 |  | .35 |
| Read about how to avoid heat stress during heat waves | .56 |  | .31 |
| Repaint your house in a brighter colour (less heat absorption in the summer) | .55 |  | .30 |
| Fit water saving device in your cistern to save when flushing | .54 |  | .29 |
| Buy a flood insurance | .48 |  | .23 |
| Kaiser-Meyer-Olkin measure of sampling adequacy | .81 |  |  |
| Bartlett's test of sphericity | *p* < .001 |  |  |
| Eigenvalue | 3.46 |  |  |
| % of variance | 43.28 |  |  |

*Notes. h^2^* = communality.
