# Supplementary material for: Hand in Hand: Public Endorsement of Climate Change Mitigation and Adaptation
Source: PLoS One. 2015 Apr 29;10(4):e0124843. doi: 10.1371/journal.pone.0124843 (PMC4414563; doi:10.1371/journal.pone.0124843)
Supplement: S6 Table — (DOCX) [file pone.0124843.s010.docx]

*S6 Table*. Summary of mitigation policy support items, factor loadings, and communalities from principal axis factor analysis.

|  | UK sample | | |  | Swiss sample | | |
| --- | --- | --- | --- | --- | --- | --- | --- |
| Item | Factor loading |  | *h^2^* |  | Factor loading |  | *h^2^* |
| Increased fuel and diesel taxes | .76 |  | .58 |  | .78 |  | .61 |
| Congestion charging on busy roads | .74 |  | .55 |  | .67 |  | .45 |
| Air travel taxation (e.g., on ticket prices) | .72 |  | .52 |  | .67 |  | .44 |
| Increasing general taxation to pay for public transport | .69 |  | .48 |  | .66 |  | .43 |
| Increased household electricity taxes | .68 |  | .46 |  | .67 |  | .45 |
| Ban the driving of cars in certain areas | .67 |  | .45 |  | .60 |  | .36 |
| Information campaigns about negative climate effects caused by car and aeroplane travel | .66 |  | .44 |  | .63 |  | .40 |
| Ban the production of vehicles with gas / fuel mileage below 75 miles per gallon | .63 |  | .40 |  | .59 |  | .35 |
| Tax for the protection of tropical rain forests | .63 |  | .39 |  | .60 |  | .36 |
| Introducing labels stating carbon content | .55 |  | .30 |  | .63 |  | .39 |
| Teach children about the causes, consequences, and potential solutions to climate change | .54 |  | .29 |  | .60 |  | .36 |
| Subsidies for electric (emission-free) vehicles | .50 |  | .25 |  | .39 |  | .15 |
| Subsidies for house insulation | .42 |  | .17 |  | .40 |  | .16 |
| Subsidies for the household production of green energy | .41 |  | .17 |  | .47 |  | .22 |
| Kaiser-Meyer-Olkin measure of sampling adequacy | .93 |  |  |  | .91 |  |  |
| Bartlett's test of sphericity | *p* < .001 |  |  |  | *p* < .001 |  |  |
| Eigenvalue | 6.02 |  |  |  | 5.71 |  |  |
| % of variance | 42.98 |  |  |  | 40.80 |  |  |

*Notes. h^2^* = communality.
