# Supplementary material for: Hand in Hand: Public Endorsement of Climate Change Mitigation and Adaptation
Source: PLoS One. 2015 Apr 29;10(4):e0124843. doi: 10.1371/journal.pone.0124843 (PMC4414563; doi:10.1371/journal.pone.0124843)
Supplement: S7 Table — (DOCX) [file pone.0124843.s011.docx]

*S7 Table*. Summary of adaptation policy support items, factor loadings, and communalities from principal axis factor analysis.

|  | UK sample | | |  | Swiss sample | | |
| --- | --- | --- | --- | --- | --- | --- | --- |
| Item | Factor loading |  | *h^2^* |  | Factor loading |  | *h^2^* |
| Obligatory integration of climate risk and adaptation assessments into business planning | .69 |  | .48 |  | .55 |  | .31 |
| Protection and creation of wetlands (improves flood protection and contributes to biodiversity) | .68 |  | .46 |  | .43 |  | .19 |
| Introduce building codes to make houses more thermally comfortable with longer and hotter summers | .65 |  | .43 |  | .38 |  | .14 |
| Train Health Services staff to identify and advise on heat stress risks | .64 |  | .42 |  | .50 |  | .25 |
| New spatial planning to reduce the risk of flooding | .64 |  | .41 |  | .56 |  | .31 |
| Creation of habitat corridors for animals (e.g., bridges over motorways) | .62 |  | .39 |  | .46 |  | .22 |
| Reduce pressure on systems or areas at risk (e.g., fewer fishing and hunting licenses) | .62 |  | .39 |  | .47 |  | .22 |
| Relocation of dwellings away from flood-prone areas | .59 |  | .34 |  | .52 |  | .27 |
| Requirement to fit houses with water resistant door and window frames in flood risk areas | .55 |  | .30 |  | .43 |  | .18 |
| Produce and distribute guidance on how to avoid heat stress | .54 |  | .29 |  | .63 |  | .40 |
| Increase national development assistance to help developing countries to adapt to climate change | .53 |  | .28 |  | .58 |  | .33 |
| Restricted hose use during the summer | .52 |  | .28 |  | .43 |  | .18 |
| Upgrade all flood defences to a higher standard | .49 |  | .24 |  | .37 |  | .14 |
| Tax to establish a fund to alleviate unavoidable climate change impacts in the UK | .48 |  | .23 |  | .54 |  | .29 |
| Close access to vulnerable places, including some recreation areas, marinas, and hiking trails | .47 |  | .22 |  | .51 |  | .26 |
| Kaiser-Meyer-Olkin measure of sampling adequacy | .92 |  |  |  | .84 |  |  |
| Bartlett's test of sphericity | *p* < .001 |  |  |  | *p* < .001 |  |  |
| Eigenvalue | 5.80 |  |  |  | 4.41 |  |  |
| % of variance | 38.65 |  |  |  | 29.42 |  |  |

*Notes. h^2^* = communality.
